# *mouseTube* – a database to collaboratively unravel mouse ultrasonic communication

**DOI:** 10.12688/f1000research.9439.1

**Published:** 2016-09-16

**Authors:** Nicolas Torquet, Fabrice de Chaumont, Philippe Faure, Thomas Bourgeron, Elodie Ey

**Affiliations:** 1 Université Pierre et Marie Curie Paris 06, CNRS UMR 8246, INSERM U1130, Institut de Biologie Paris-Seine (IBPS), Sorbonne Universités, Paris, 75005, France; 2 Bio Image Analysis, CNRS URA 2582, Institut Pasteur, Paris, 75015, France; 3 Human Genetics and Cognitive Functions, CNRS UMR 3571 Genes, Synapses and Cognition, University Paris Diderot, Sorbonne Paris Cité, Institut Pasteur, Paris, 75015, France

**Keywords:** Mouse, ultrasonic vocalisations, mouse communication, database, mouseTube, mouse behaviour, open data, open analysis

## Abstract

Ultrasonic vocalisation is a broadly used proxy to evaluate social communication in mouse models of neuropsychiatric disorders. The efficacy and robustness of testing these models suffer from limited knowledge of the structure and functions of these vocalisations as well as of the way to analyse the data. We created *mouseTube*, an open database with a web interface, to facilitate sharing and comparison of ultrasonic vocalisations data and metadata attached to a recording file. Metadata describe 1) the acquisition procedure, *e.g*., hardware, software, sampling frequency, bit depth; 2) the biological protocol used to elicit ultrasonic vocalisations; 3) the characteristics of the individual emitting ultrasonic vocalisations ( *e.g.*, strain, sex, age). To promote open science and enable reproducibility, data are made freely available. The website provides searching functions to facilitate the retrieval of recording files of interest. It is designed to enable comparisons of ultrasonic vocalisation emission between strains, protocols or laboratories, as well as to test different analysis algorithms and to search for protocols established to elicit mouse ultrasonic vocalisations. Over the long term, users will be able to download and compare different analysis results for each data file. Such application will boost the knowledge on mouse ultrasonic communication and stimulate sharing and comparison of automatic analysis methods to refine phenotyping techniques in mouse models of neuropsychiatric disorders.

## Introduction

Mice are the most broadly studied animal models in scientific research. They are used to understand causes and mechanisms of human diseases, as well as to develop new therapeutic strategies. More and more scientists are interested in their social behaviour, and aim to improve housing conditions, better understand the way pharmacological substances or genetic mutations act on social life, or simply better know their models in order to develop the most adequate tests.

Mice are social animals and use olfactory, tactile, visual but also auditory signals to regulate their relationships. Indeed, mice emit audible and ultrasonic vocalisations to communicate with their conspecifics. These vocalisations might represent an “easy-to-record” proxy for sociality. Audible signals (20 Hz – 20 kHz) are much less frequent than ultrasonic vocalisations; we therefore focus on ultrasonic vocalisations, ranging between 20 kHz and more than 120 kHz. Mouse ultrasonic vocalisations are rapidly successive pure tones of short duration, high frequency modulations, and with or without frequency jump(s) (reviewed in ( Portfors, 2007)). Pups utter isolation calls in their first 2 weeks of life ( Zippelius & Schleidt, 1956). These vocalisations reliably trigger maternal retrieval ( Sewell, 1970; Zippelius & Schleidt, 1956). Juvenile and adult mice utter ultrasonic vocalisations when encountering an unknown conspecific of the same sex ( Chabout *et al.*, 2012; Hammerschmidt *et al.*, 2012; Maggio & Whitney, 1985; Panksepp *et al.*, 2007). Both sexes utter these calls, but male vocal behaviour is maximised by social isolation in many cases ( Chabout *et al.*, 2012; Scattoni *et al.*, 2010). These vocalisations may play a role in social recognition and hierarchy establishment, at least in females ( D’Amato & Moles, 2001; Moles *et al.*, 2007). Finally, sexually mature males vocalise when encountering an oestrus female or urinary cues from her ( Holy & Guo, 2005; Whitney *et al.*, 1973). These calls increase the probability of the female staying in proximity with the male emitter ( Hammerschmidt *et al.*, 2009).

Despite these identified contexts of emission, knowledge about the real significance and structure of mouse ultrasonic vocalisations still suffers from several weaknesses. First, the functions of these vocalisations, specifically those emitted by juvenile and adult mice, are still unclear. Indeed, male calling in presence of an oestrus female might represent a courtship situation ( Holy & Guo, 2005). These calls are nevertheless not structurally different from those of adult females, suggesting at least another function such as proximity maintenance ( Hammerschmidt *et al.*, 2012; Seagraves *et al.*, 2016). It is also still unclear to what extent the temporal organisation and the fine acoustic structure of the calls are meaningful for the receiving mice. They are physiologically able to perceive subtle acoustic variations ( Portfors *et al.*, 2009), but behavioural evidence for the meaning of these subtle variations remains scarce ( Hammerschmidt *et al.*, 2009; Wöhr *et al.*, 2011a). Second, the emission of ultrasonic vocalisations is highly dependent on the emitter’s identity ( Holy & Guo, 2005), the receiver’s identity ( Seagraves *et al.*, 2016), and the context ( *e.g.*, Yang *et al.*, 2013). These sources of variability remain under-explored and could explain the lack of reproducibility in several assays. Finally, the domain suffers from a lack of automation of the analysis of these signals. Some laboratories have developed their own detection and/or analysis methods ( *e.g*., Chabout *et al.*, 2015; Hammerschmidt *et al.*, 2012; Holy & Guo, 2005; Neunuebel *et al.*, 2015; Seagraves *et al.*, 2016; von Merten *et al.*, 2014) or use commercial and/or manual solutions for detection and/or analysis ( Chabout *et al.*, 2012; Ey *et al.*, 2013; Wöhr *et al.*, 2011b; Yang *et al.*, 2013). Nevertheless, little is known about the advantages and disadvantages of each of these methods, and comparisons of these methods on the same files would be highly valuable for the field.

To counteract these current weaknesses of the domain, we developed *mouseTube*. This database is designed to share and exchange recording files from mouse ultrasonic vocalisations, along with all the corresponding metadata. It aims at increasing knowledge on mouse vocal communication, improving reproducibility of the experiments and stimulating the development of robust analysis tools. The web interface is available at http://mousetube.pasteur.fr.

Data uploaded on *mouseTube* are shared between all members of the community connected to *mouseTube*. Members uploading data on *mouseTube* are fully responsible for the content of uploaded files and the accuracy of the metadata provided. Data uploaded still belong to the laboratory that recorded them, but the owner gives the right to the members of *mouseTube* to use them for analyses and publications. Any member of the community can download data. *mouseTube* data are freely available upon online registration and can be used for subsequent publications. Any publications derived from the data should state the contributors (user) of the data and mousetube.pasteur.fr as being the data source and, whenever possible, cite the original paper(s) in which data have been first described. *mouseTube* administrators decline all responsibilities for the content of data and metadata.

## Data and metadata

### Database design

The *mouseTube* database stores the links toward each audio recording file and all the corresponding metadata. The audio recording files themselves are stored on external servers, owned by each laboratory and accessible with the login and password given when registration is confirmed (these servers should be configured with these login and password). This allows the owner to control his or her own data. For a video tutorial on how to upload data on mouseTube, please see ( Ferhat *et al.*, 2016).

*mouseTube* is a web interface coded in php and a MySQL relational database hosted by an Apache server. The web interface allows users ( *i.e.*, data contributors and data downloaders) to manage the data in the database. In relational databases, each table has its own unique key to connect tables together (it is possible to combine several keys but in the case of *mouseTube*, we identify a simple unique key for each table). In this way, the data are well organised and it is very fast to find all the specificities of an element, looking through the links.

**Figure 1.**
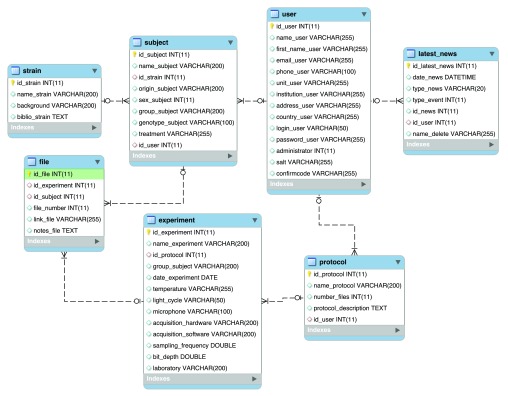
Overall organisation of the *mouseTube* database. *mouseTube* is organised in 7 tables connected by unique keys.

*mouseTube* is organised as seven tables connected by unique keys within the database ( Figure 1):

- “strain”: gathers all mouse strains already entered in the database. This table contains information about the name of the strain, the background on which it has been generated, and the bibliographic reference where it has been first described. If a new strain is needed, every user can send an email with all requested information for the administrators to add it. This procedure will avoid any.
- “subject”: lists all the individual mice entered by contributing users. New subjects are created by each contributing user. The table “subject” is connected to the table “user” (unique key “id_user”), meaning that one individual belongs to only one contributing user. The “subject” table is also connected to the table “strain” by the unique key “id_strain”, meaning that one subject can only be characterised by one strain. This table stores all information relative to each subject such as its origin, name, sex, genotype, treatment, and subgroup.
- “user”: stores the information about all contributing and non-contributing users having access to the *mouseTube* database. This table is connected to the table “subject” and “protocol” through the unique key “id_user”, identifying the owner of a subject and the protocols that have been used by this person (a subject or a protocol belongs to only one user). The table “user” stores contact information and the encrypted version of the password and login. All user information can be changed by him/herself whenever he/she needs to.
- “protocol”: lists all the protocols entered by users. Protocols are created as free text by each user and should provide enough information to be replicable. This table is connected to the table “user” through the unique key “id_user” designating its creator. The table “protocol” contains information about the name of the protocol, its description, which user has created it, and the number of recording files generated for each mouse with this protocol.
- “experiment”: lists all the experiments ( *i.e*., a set of audio files recorded for a group of individuals with one protocol) entered by the users. New experiments can be created by each user. It is connected to the “protocol” table through the unique key “id_protocol” since each experiment involves one unique protocol.
- file: lists all the vocalisations files entered by the users, for each individual mouse (unique key “id_subject”) within each experiment (unique key “id_experiment”). This means that one file relates to a unique subject and a unique experiment. The preferred format is the uncompressed “.wav” one.
- “latest news”: this table retraces all the new actions that have been performed on *mouseTube*. It allows researchers to follow updates made by each user (unique key “id_user”).

### Data organisation

A help section is available on the home page of each user’s session. This part provides all details about the information stored in each section of *mouseTube*. It can also be accessed at any time through the “Help” tab available on each page.

For each protocol, a name is requested. Users should also specify how many recording files are generated for each mouse in this protocol. Users should then provide a very precise description of the exact protocol. If another user has already created the protocol, the owner of the vocalisation files needs to create it again with a similar name and same information. He/she will then be able to select it when he/she creates an experiment. The database is built in this way to track changes in each protocol.

For each experiment embedding several audio recording files, the following metadata are stored:

- name of the experiment
- protocol
- mouse group
- date of the beginning of the experiment
- temperature
- light phase for testing (light or dark period)
- hardware used (microphone & sound card; auto-completion of this field to ease the identification of the equipment)
- acquisition software
- sampling frequency
- bit depth
- laboratory

These metadata will permit useful comparisons of the software, hardware and environmental conditions of each audio recording. This information is compulsory to evaluate constraints on each recording.

### Data retrieval

The *mouseTube* web interface allows users to search different types of data into the database without coding a MySQL request. Data can be searched according to several criteria, such as protocols, owner of the files, mouse strain, and experiment. Users can select all recording files from a specific mouse strain or user/laboratory. *mouseTube* also aims at providing the users with different protocols to record mouse ultrasonic vocalisations. *mouseTube* even provides the possibility to search for individual mice. The more information is entered, the easier it is to find a subject. To download any vocalisation file, users need the login and password provided at the confirmation of their registration and common to all servers hosting vocalisation files linked in *mouseTube*. These are different from their personal login and password.

## Use cases

### Example 1: searching for protocols and control strains

In this example, we use the search function of *mouseTube* in the protocol section to gather information on potential protocols to record ultrasonic vocalisations in adult male mice and to collect data on control animals in this protocol.

**Figure 2.**
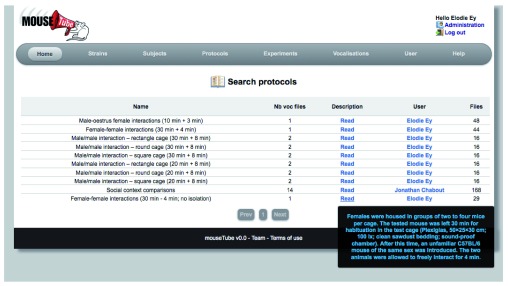
Screenshot of the *mouseTube* web application on the search page for protocols.

By going through all protocols ( Figure 2), we found several protocols used to record male ultrasonic vocalisations. Elodie Ey provided protocols to record male-male interactions in different cages after 3 weeks of isolation or male-female interactions. Jonathan Chabout also provided a protocol to record male vocalisations in response to urine (male or female), an anesthetised mouse (male or female), or an active female. With these selected protocols, at the time of writing, we managed to find 16 individuals from the C57BL/6J strain recorded in the male-male interaction protocol from E. Ey, 48 individuals from the *ProSAP1/Shank2* strain recorded in the male-oestrus female protocol from E. Ey, and 12 individuals from the B6D2F1/J strain recorded with the protocol of J. Chabout. Altogether, we now have an important set of reference files for each protocol to compare to our data, and soon we will try the different protocols in our laboratory. The output file (Supplementary file 1) provides a sample of the metadata and links to the files recorded in the male – oestrus female interactions by E. Ey. This file is automatically generated for each vocalisation search request and can be downloaded to save the metadata related to each file.

Output file automatically generated for each request of vocalisation file A sample of the metadata and links to the files recorded in the male – oestrus female interactions by E. Ey. This file is automatically generated for each vocalisation search request and can be downloaded to save the metadata related to each file. Click here for additional data file. Copyright: © 2016 Torquet N et al. 2016 Data associated with the article are available under the terms of the Creative Commons Zero "No rights reserved" data waiver (CC0 1.0 Public domain dedication).

### Example 2: testing a detection algorithm

In this example, we plan to test a new detection algorithm under various recording conditions. To do so, we need to gather files recorded with different equipment under various levels of background noise conditions. We use the search function of *mouseTube* in the vocalisation section to select three vocalisation files in each of the protocols available in *mouseTube*. The diversity of recording conditions allows us to investigate the limit of our detection algorithm to extract ultrasonic vocalisations from background noise. We end up with a set of many vocalisation files to include in our test set. For each vocalisation search, a table recapitulates the corresponding metadata of each vocalisation file, specifying the protocol and hardware and software used. We can therefore test our detection algorithm on files knowing the number of individuals present during the recording session and the background noise that is then generated as well as the quality of the recording equipment ( *e.g*., the microphone frequency response).

## Conclusion

We present *mouseTube*, a database with a web application to boost knowledge on mouse ultrasonic communication. This database stores recording files of mouse ultrasonic vocalisations as well as the corresponding metadata. It provides a source of information on the protocols to record mouse ultrasonic vocalisations and on the availability of recording files for different mouse strains.

At the time of writing this paper, *mouseTube* provides a platform to up- or download mouse recording files and the corresponding metadata. The aim is to constantly develop *mouseTube*, and we are currently exploring ways of enabling users to analyse their data online, where the owner of the data will be notified of each analysis performed with their data. The database will also offer researchers the option of keeping a portion of their data on a private part of *mouseTube* until they have been analysed and published, after which the data will be made publicly available. We will develop this analysis system shortly. We also aim to open *mouseTube* to plug-in other analysis systems. The users will then be able to choose which software they want to analyse their data.

## Data and software availability

Audio recording files are available for all *mouseTube* users via the web application http://mousetube.pasteur.fr. To download the vocalisation files from the different storage servers, users need to enter the login and password common to all servers hosting *mouseTube* data files. These are provided upon registration online.

F1000Research: Dataset 1. Output file automatically generated for each request of vocalisation file, 10.5256/f1000research.9439.d135667 ( Torquet *et al.*, 2016).
