# Supplementary material for: mouseTube – a database to collaboratively unravel mouse ultrasonic communication
Source: F1000Res. 2016 Sep 16;5:2332. [Version 1] doi: 10.12688/f1000research.9439.1 (PMC5081159; doi:10.12688/f1000research.9439.1)
Supplement: Peer review history

# Peer review: Referee response for version 1

**DOI:** 10.5256/f1000research.10167.r16373

**Published:** 2016-10-21

**Authors:** A. Katrin Schenk

**Affiliations:** 1 Department of Physics, Randolph College, Lynchburg, VA, USA

This article reports about an open mouse ultrasonic vocalization (USV) database (mouseTube) to store both USV data and its metadata about recording equipment, conditions and experimental protocols. As the authors clearly point out, the field of mouse ultrasonic vocalization research is challenging due to the very high variability of both the data itself and the experimental protocols, recording equipment and analysis software/approaches used. The establishment of the mouseTube database, if implemented well and adopted by mouse USV researchers, could represent the single most important advance in the field to date. mouseTube has the potential to enhance experimental reproducibility, standardize recording condition reporting and allow for a rich test bed for analysis software and methods. Again, this will SIGNIFICANTLY advance the field. 

 As for implementation, the article gives detailed information on the structure of the mouseTube database and several examples of specific use cases. The structure is well thought out and it is clear that, as more users come to mouseTube, the database will be flexible enough to accommodate iterative user-driven changes. 

 Overall, the article describes a well thought out implementation of a desperately needed tool for USV research.

I have read this submission. I believe that I have an appropriate level of expertise to confirm that it is of an acceptable scientific standard.

# Peer review: Referee response for version 1

**DOI:** 10.5256/f1000research.10167.r16689

**Published:** 2016-09-30

**Authors:** Takefumi Kikusui

**Affiliations:** 1 Department of Animal Science and Biotechnology, Azabu University, Sagamihara, Japan

This paper is presenting the web-based mouse ultrasonic vocalization database. The aim and the methods of the system MouseTube are well designed and organized. My only concern is with how many users would be registered and how many calls would be uploaded. More users, more fascinating. Therefore the authors need to do other things to collect the users and calls. For example, DNA seq data should be registered if papers using the new sequence need to register the sequence first before paper submission. A such “obligated” system would accelerate the values of this system. If the authors can propose such things to the scientific committee, that would be fantastic.

 I believe that both the title and abstract are appropriate for the content of the article. The design, methods and analysis of results from the study have been explained and are appropriate for the topic studied. The conclusions are sensible, balanced and justified on the basis of the results obtained.

I have read this submission. I believe that I have an appropriate level of expertise to confirm that it is of an acceptable scientific standard.

# Response

**Published:** 2016-10-20

**Authors:** Elodie Ey

**Affiliations:** CNRS - Institut Pasteur, France

We thank the reviewer for his comment. We agree that it would be helpful to broaden the distribution of the system. We are working on it. We are working on a way to couple the analysis of the data files with the database. This will for sure attract many users. More and more journals ask for the availability of the raw data, and we should make sure that mouseTube will be accepted as a way to publish the raw data. Some journals accept it; others are still reticent. We are also working on it.
